# On the Diagnosis of Fracture

**Published:** 1845-12

**Authors:** 


*On the Diagnosis of Fracture.*—By Mr. Grantham.*—Although the following method is by no means new to the English

*Facts and Observations on Medicine and Surgery. London, 1845. p. 61

surgeon, it is at present but seldom adopted. "The stethoscope applied over the place of fracture, in the slightest motion of the part, conveys a much more decided crepitus than is perceived by the naked ear during the most extended movements of the part. In many cases, even the slight pressure of the ear on the stethoscope, suffices to produce the crepitation, a circumstance of no small importance, as freeing the patient from the pain unnecessarily excited by the motion requisite in the manual examinations. The crepitus yielded by the more solid bones is sonorous, and resembles the sound produced by breaking a piece of wood across the knee; it is accompanied with a sensation of roughness unpleasant to the ear. The sound yielded by the more spongy bones is duller, and resembles the effect of a rasp on wood; except that now and then this noise is broken by a sound of a clearer kind, like those afforded by the compacter bones. only not so loud. The sound from oblique fractures is stronger than from those which are transverse; but when one end of the fractured bone rides over the other, the sound is then obscured, and, in some cases, may not be perceived without slight extension or counter-extension of the limb. If the fracture is comminuted, the sensation, as of distinct portions of the bone, is conveyed by the stethescope. When fluids are effused around the fracture, a gurgling is combined with the crepitation, and which is compared to the sound produced by a shoe full of water." (Lisfranc.) A dry crepitus rattle is produced by inflammation of the cellular structure, wherein the serum becomes suppressed, and the cells distended with air, which may be mistaken for the crepitus arising from fracture. It is much louder, and may be distinctly heard by the patient or bystander; it is heard by making gentle pressure with the fingers, or end of the stethescope, over the injured part; it is most distinct on the third day, and decreases on the fifth; it is a sound apt to be mistaken for fracture of the fibula.—We have a sound like this in the common subcutaneous emphysema, on pressing uninterruptedly with the hand on the affected part.—*Ibid.*

